# On the definition of noise

**DOI:** 10.1057/s41599-022-01431-x

**Published:** 2022-11-08

**Authors:** Fangfang Liu, Shan Jiang, Jian Kang, Yue Wu, Da Yang, Qi Meng, Chaowei Wang

**Affiliations:** 1 Heilongjiang Cold Region Architectural Science Key Laboratory, School of Architecture, Harbin Institute of Technology, 66 West Dazhi Street, Nan Gang District, 150006 Harbin, PR China; 2 Institute for Environmental Design and Engineering, University College London, London, WC1H 0NN UK

**Keywords:** Environmental studies, Science, technology and society

## Abstract

Urbanization has exposed people to extreme sound levels. Although researchers have investigated the ability of people to listen, analyze, and distinguish sound, the concept of noise has not been clearly articulated from a human perspective. The lack of knowledge on how people perceive noise limits our capacity to control it in a targeted manner. This study aimed to interpret the definition of noise from the public perspective based on a grounded theory approach. Seventy-eight participants were interviewed about noise, and four categories of perceived understanding of noise were identified: challenges, definitions of noise, opportunities, and action. As one of the challenges, urbanization is associated with increased noise levels around the human environment. In terms of definition, perceiving sound as noise is considered to be a result of the complex and dynamic process that includes sound, the environment, and humans. Sound and humans interact with the environment. In terms of opportunities, noise may have positive roles on certain occasions, dispelling the misconception that noise is exclusively negative. In addition, we found that noise perception has gradually shifted from noise control to noise utilization. In terms of action, noise can be controlled at the sound sources, susceptible target groups, susceptible behaviors and states, locations, and times where noise is perceived with high frequency. In this study, we investigated several aspects of noise, ranging from noise control, soundscape definition, and ‘soundscape indices’ (SSID) integration and application. Our findings provide an additional basis for developing better definitions, control, and utilization strategies of noise in the future, thereby improving the quality of the sound environment.

## Introduction

Noise has plagued mankind and has been studied for many years. The first ever complaint about noise was recorded in the second millennium BC as part of the Mesopotamian epic Atrahasis, which refers to noise disturbance depriving people of sleep. Noise, a common law nuisance, was reported in England in the 18th century and included the continuous ringing of church bells (day and night), which severely disrupted people’s sleep. Besides, the industrial revolution introduced industrial machinery noise, and the rapid growth of many cities around the Second World War enhanced noise problems. In addition, the concept of the soundscape was reported (Schafer, 1996). It is important to generate good sound but control noise (Kang, 2019). Murray R. Schafer wrote in his book *The Soundscape: Our Sonic Environment and the Tuning of the World* (Schafer, 1996), “Starting with nature’s primal sounds, humans have encountered this ever complexity of our acoustic surrounds. As humanity progresses, new sounds emerge all around us. Currently, massive acoustic information is available which reduces our ability to listen to the complexities and intricacies of sound.” He claims that our goal is to hear, analyze, and create distinctions. The International Organization for Standardization defines a soundscape as, “acoustic environment as perceived or experienced and/or understood by people, in context” (Standardization IOf, 2014). Although the standards and controls for noise exposure have improved, the noise problem remains inadequately addressed in some countries (Silva et al., 2020; Yongbing and Hal Martin, 2013). Noise is a major public health issue that continues to grow (WHO, 2018). Thus, the reduction of noise levels is a key focus of global environmental acoustics regulations and policies and the soundscape field (Kang, 2019). Since professional and lay people have divergent understandings of the acoustic environment, conflicts can occur when the public’s risk perception differs from that of government experts (Liu et al., 2021; Yang, 2020). Therefore, noise control strategies should consider the needs of all stakeholders, which include the public. In fact, involving the public in the decision-making processes is effective in the implementation of noise management interventions (Heyes et al., 2021; Riedel et al. 2021a, 2021b). Therefore, it is important to understand noise from a public perspective.

### Literature review

A bibliometric approach based on VOSviewer (v.1.6.17) and CiteSpace (v.5.7 R5) analysis were adopted in this study. Bibliometric analysis monitors changes in specific research areas from a quantitative perspective thereby revealing emerging trends and research progress in specific areas. Bibliometric analysis is often performed in conjunction with visualization using charts created through VOSviewer and CiteSpace. These charts allow for a clear and effective presentation of data. A title search was conducted on the Web of Science using the keyword ‘noise’ to identify studies published between 2010 and 2022. A total of 20,072 studies were obtained. The studies were collected and analyzed using data mining visualization software VOSviewer and CiteSpace to identify research hotspots and co-category networks.

**Fig. 1:**
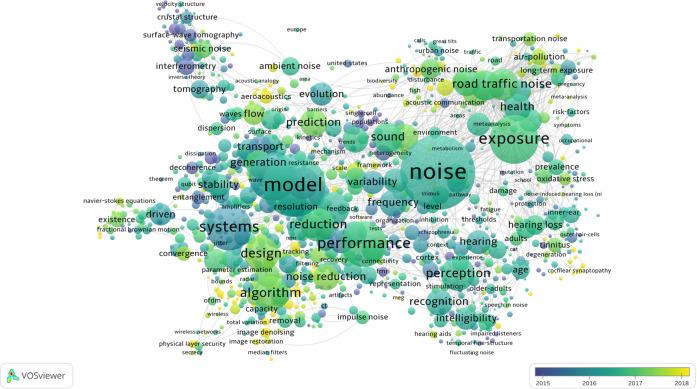
Visualization of research hotspots.

In Fig. 1, the purple to the blue region represents research hotspots in the field of noise research from 2015 to 2016, which mainly include surface-wave tomography, interferometry, single-cell, populations, decoherence, transport, heterogeneity, systems, evolution, tomography, and variability. The blue to green region indicates the research hotspots between 2016 and 2017 which mainly include health, perception, ambient noise, recognition, exposure, hearing loss, environment, seismic noise, design, algorithm, prediction, tracking, noise reduction, anthropogenic noise, transportation noise, air pollution, and road traffic noise. Finally, the green to yellow region denotes the relevant research hotspots between 2017 and 2018, which include aeroacoustics, image denoising, physical layer security, and cochlear synaptopathy.

In summary, research on noise covers various aspects including noise (2015–2016, purple to the blue band), the relationship between noise and humans, noise classified according to different environments, noise prediction and control (2016–2017, blue to green band), and noise control design at the technological level. Research hotspots appear to be in highly sophisticated fields such as aerospace and medicine (2017–2018, green to yellow range) (Fig. 1).

**Fig. 2:**
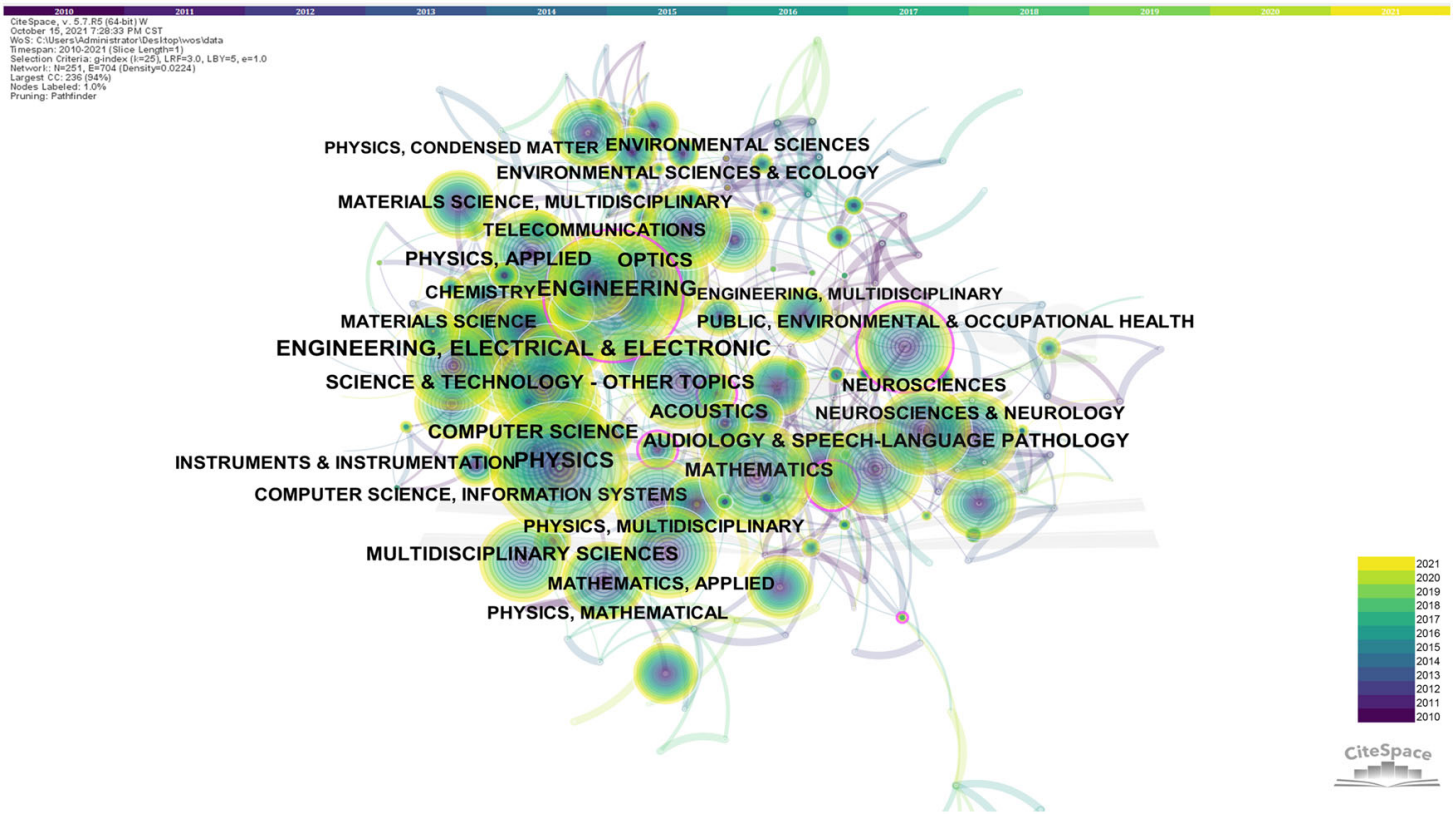
Co-category network analysis.

In CiteSpace, time was set to 2010–2022, with a one-year time slice, yielding a total of 251 nodes and 704 connected lines. The results of the co-category network analysis were visualized in the form of a visual network (Fig. 2). The size of each node reflects the co-occurrence frequency of the subject categories. In CiteSpace, centrality is a metric that finds and measures the importance of a document in the network, with values ranging from 0 to 1. Literature with high centrality (centrality ≥ 0.1, nodes with purple rings) is often a key hub connecting two different domains. Six subject categories including Environment Science & Ecology (0.18), Psychology (0.14), Neuroscience & Neurology (0.14), Computer Science, Interdisciplinary Application (0.11), Linguistic (0.10), and Public, Environmental & Occupational Health (0.10) had a centrality ≥ 0.1.

In Environment Science & Ecology, noise is defined as any acoustic phenomenon that causes an unpleasant or disturbing sensation (Australia, 1997; Can et al., 2020). In this context, noise is considered to have detrimental effects on the environment, the soundscape, and disrupt the natural and ‘necessary’ quietness (Thompson, 2017). High noise levels are the most challenging to describe and mitigate despite the growing desire of urban dwellers for a comfortable and environmentally friendly city (Can et al., 2020; Organization, 2011).

In psychology, noise is a genome-wide phenomenon comprising intrinsic and extrinsic noise induced by the stochastic nature of biochemical reactions and the microenvironment, respectively (Zhou et al., 2022). Each of the Big Five personality dimensions (neuroticism, extroversion, openness, agreeableness, and conscientiousness) affects human perception of noise sensitivity (Shepherd et al., 2015). Extroversion and neuroticism are the most important factors influencing noise sensitivity and noise annoyance when compared with conscientiousness and openness to experience (Moghadam et al., 2021).

In Neuroscience and Neurology, random disruptions of signals, which are referred to as ‘noise,’ pose a basic problem in the processing of information and affect every aspect of nervous-system features (Faisal et al., 2008). Computational noise is defined as the random variability of model updates within the environment that can reflect true neural noise (e.g., the reward probability associated with each choice option in an inverse learning task). Even though computational noise is one of the most common causes of inference errors in perceptual decision-making, it results in less predictable behavior, which is beneficial in competitive social contexts.

In Computer Science, Interdisciplinary Application, noise is defined as an unwanted disturbance in an electrical signal. Besides, noise has a loop-breaking effect on the stability of network systems (Guo et al., 2021). Real-world data is imperfect and is often prone to corruption (noise) that can affect data interpretation, model creation, and decision-making (Zhu and Wu, 2003).

In Linguistics, noise is defined as sounds that corrupt the recognition of spectral information in speech (Chen et al., 2014; Fu et al., 1998). Research on the effects of noise on human conversation comprehension, speech perception, and spoken language recognition has shown that older listeners are more likely to experience difficulties in understanding speech, especially in challenging environments with background noise (Ben et al., 2011). Background noise affects the number of correctly recognized words, in addition to reaction time and perceptual hearing (Larsby et al., 2008).

Many studies in Public, Environmental & Occupational Health have investigated the association between environmental noise, noise annoyance, and potential effects (e.g. health effects, work performance, psychological state, and cognitive performance) (Xing et al., 2021). Noise has been found to trigger worry-related negative emotional reactions such as irritability, distress, and fatigue brought. Dissatisfaction with the sound environment can have a negative impact on health, well-being, job satisfaction, productivity, and many other life aspects (Babisch et al., 2009; Basner et al., 2014; Xing et al., 2021). Noise has an economic impact, with noise pollution costing 0.2–2% of GDP, and house prices fall by 0.6% for every decibel increase in noise (Kang, 2007).

### Aim and objectives

There has been no systematic research on what noise is, how people perceive it, and if noise is perceived differently by the general public and professionals. This study employed a user-centered systematic qualitative analysis of noise and grounded theory interview methods. It aimed to understand how noise is understood and defined from the basis of general public perception using interview data.

## Methods

Quantifiable research is widely used in the social sciences and humanities, including anthropology, sociology, education, health sciences, history, and so on. Quantifiable research can be used to gain in-depth information and insight into a problem as well as to generate new research ideas (Acun and Yilmazer, 2018, 2019; Conrad, 1978; Glaser, 1992, 2002; Glaser et al., 1968). Accordingly, a qualitative study based on the grounded theory (GT) method of analysis was used to investigate the perceptions of people on noise.

### Participants and interviews

**Fig. 3:**
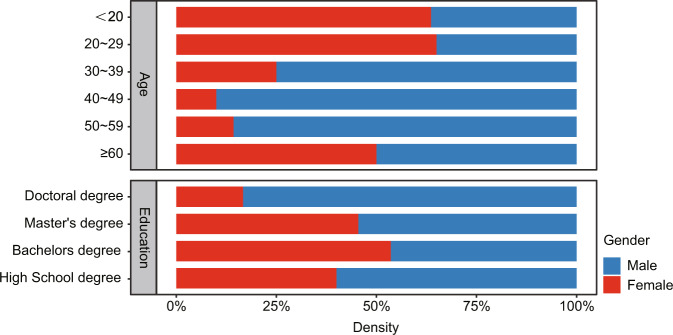
Participants’ age and education information.

In this study, we conducted semi-structured interviews through circular discussions around the concept of noise to understand the public’s general understanding of noise. At theoretical saturation, there were 78 interviewed respondents including 38 females and 40 males; ranging between 18 and 62 years old (mean age was 29.67 and the standard deviation (SD) was 12.72) (Acun and Yilmazer, 2018; Davies et al., 2022; Karimimoshaver et al., 2020; Liu and Kang, 2016; Liu et al., 2021; Lovrić et al., 2018; Pryce et al., 2018; Zhang et al., 2021). Furthermore, the respondents were from 11 daily occupations, including students, teachers, engineers, designers, civil servants, financial industry workers, workshop workers, restaurant owners, drivers, housewives, and freelancers. The research team interviewed six foreigners with a history of residence and travel to China, including two from Germany, and one from Korea, Nigeria, Poland, and the United Kingdom, to learn about how foreigners perceive noise in China. The age, gender, and education level of the interviewees are shown in Fig. 3. Ethical review and approval were waived for the study on human participants in accordance with local legislation and institutional requirements.

The interview was structured around the following two aspects: (1) demographic information, such as age, gender, occupation, and education level of the respondent and (2) the perception and opinion of people on noise, which was captured in questions such as what do you consider as noise in your understanding? Can you describe it? Can you discuss noise from the perspective of your daily life?

A total of 78 interviews, each lasting 30–60 min were conducted. With the consent of the interviewees, each interview was recorded and transcribed by the researcher, producing approximately 36,000 words of interview transcripts.

### Data analysis

The data analysis was performed by a team of two researchers who have worked in the field of soundscape and are familiar with qualitative research (Zhang et al., 2021). The two data analysts from the research teams independently coded the interview texts but were not involved in pre-programming to ensure the objectivity of the research findings. The results of the two data analysis teams were then consolidated and compared and passed to the expert panel for scrutiny (Pryce et al., 2018). The expert panel consisted of two experts working in the field of acoustics and familiar with qualitative research and assessed the credibility of the coding to ensure the accuracy of the results and the soundness of the way the data were coded (Davies et al., 2022).

**Fig. 4:**
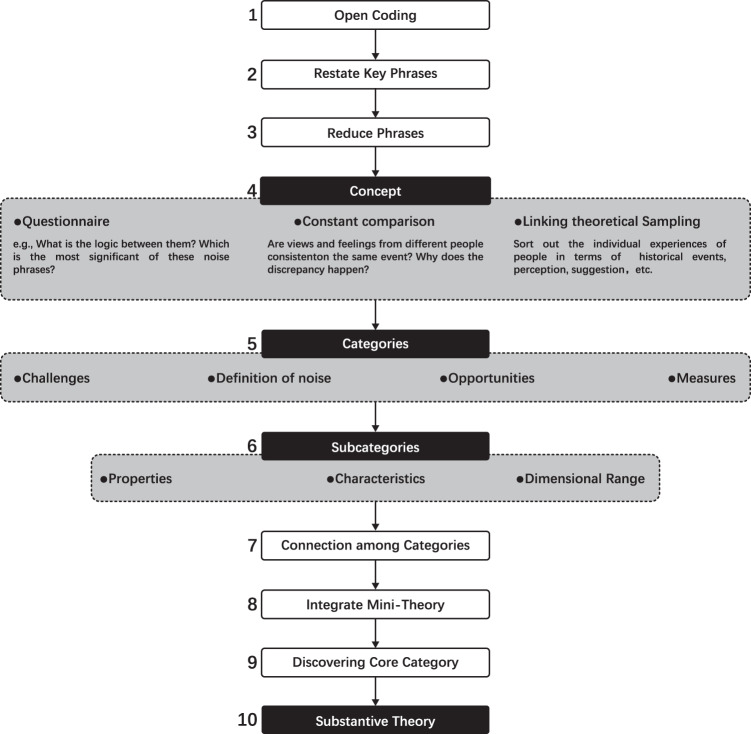
The grounded theory-based procedures.

Grounded Theory (GT) is a user-centered approach to the systematic analysis of qualitative data (Acun and Yilmazer, 2018, 2019). It can be summarized simply as “finding theory in data” (Glaser et al., 1968). The results of the multi-step analytical method based on Glaser’s methods (Glaser, 1992) are shown in Fig. 4. The procedure was as follows:

(1) *Open coding*: outlining and coding keywords in the text about the subjective perceptions of noise (Acun and Yilmazer, 2019); (2) Restating key phrases: reading relevant sections similar to the interview text and recognizing recurring words and expressions; (3) Reducing phrases: accumulation and reduction of codes; (4) Identifying concepts: posing questions, attempting to compare and relate theoretical sampling, recommending terms for concepts, and determining the logic between them. For example, identifying if any of the soundscape phrases is the most significant. Finally, the concepts were compared, and new questions were posed in light of the preliminary findings (Conrad, 1978); (5) Generating categories: Relying on a few similar concepts in the perception of participants of noise, similar concepts were combined to form categories that included noise definitions and measures of progress. Other categories were made up of various concepts that have been combined; (6) Identifying subcategories: Trying to identify subcategory characteristics and attributes across a variety of aspects; (7) Linking categories: Making associations between categories; (8) Integrating mini-theory: incorporating mini-theories to improve the application strength of the theory; (9) Discovering core categories: just like noise definitions with wide relationships to those other categories; (10) Generating the substantive theory: on noise perception and subjective perception recognizing (Glaser, 2002).

The interview text was collated and analyzed according to the aforementioned steps, resulting in 894 labeled data points (a1), 527 conceptualized data points (aa1), 67 categorized data points (a1), and four categorized data points (aa1). The specific results are shown in Table 1.

**Table 1:** Illustration of the GT-based encoding process.

Sorting memos	Labeling	Conceptualizing data	Categorizing data	Categories	Subcategorized
*What can be considered as noise according to your understanding? Can you describe it?* “Noise is the sound that is noticed, it is the noise that produces the disturbance, many sounds are not noticed and it doesn’t matter. What about noticing? There are sounds that interfere with your behavior. If you are on the phone, for example, it interferes. And when you are working, for example, it interferes. or talking, he interferes. I think it’s noise that interferes with people’s behavior or thoughts.” *Can you talk about noise in relation to your daily experience?* It’s when you want to do something, you notice the noise, and this doing may also be, for example, what is called a proper job, or it may be to find something or do something, just a behavior. It is the person who does more things, and this doing is a particularly broad definition. For example, when thinking about the sun, sunbathing, or whatever, or she also needs to be quiet for a while, he needs to rest and relax, for example, with his eyes closed, when all the external things are all noise. So it’s just I think it’s still just about the behavior. *What can be considered noise according to your understanding? Can you describe it?* Noise is the sound that one does not want to hear. Anything that is unpleasant to the ear is. An environment in which all kinds of unpleasant sounds persist is called a noisy environment. *Can you talk about noise in relation to your daily experience?* The definition of noise is also determined by the mood of the person. When people are depressed and weak, they define a large number of sounds as noise. The presence of noise is most likely to be perceived when people are in a depressed mood. …	a1 Noise is the sound that is noticed, it is the noise that produces the disturbance, many sounds are not noticed and it doesn’t matter. a2 There are sounds that interfere with your behavior. a3 Noise can interfere when on the phone a4 Noise interferes when you’re working a5 Noise interferes when talking a6 It is what is noticed, what interferes with human behavior or thought, that I think is noise a7 It’s when you want to do something, you notice the noise, and this doing may also be, for example, what is called a proper job, or it may be to find something or do something, just a behavior. a8 It is the person who does more things, and this doing is a particularly broad definition. a9 For example, when thinking in the sun, sunbathing or whatever, or she also needs to be quiet for a while, he needs to rest and relax, for example, with his eyes closed, when all the external things are all noise a10 So it’s just I think it’s still just about the behavior. a11. Noise is the sound that one does not want to hear. Anything that is unpleasant to the ear is. a12. An environment in which all kinds of unpleasant sounds persist is called a noisy environment. a13. The definition of noise is also determined by the mood of the person. a14. When people are depressed and weak, they define a large number of sounds as noise. a15. The presence of noise is most likely to be perceived when people are in a depressed mood..…	aa1 Noise exists all the time in human daily life. (a344,a351,a555 and a635) aa2 Noise is everywhere in our daily lives (a280, a479, a512, and a720) aa3 Noise is inevitable in our daily lives (a207, a558, a651, and a795) aa4 A car horn is nothing more than a necessity, and a small vendor just wants to sell things in the street. (a840) aa5 There is nothing you can do with children running around upstairs in your house and you want to sleep, there is nothing anyone can do to change the reality of this (a847) aa6 The sudden noise in a quiet coffee shop, which sadly is the norm for people, makes one wonder about the level of upbringing. (a408) aa7 I think that a lot of noise is caused by a lack of public spirit. It’s not that you don’t realize you are making noise, it’s that you don’t have the health and feelings of others at heart. (a466) aa8 It seems that noise is also related to the quality of people. (a656) aa9 It’s probably because no one is educated that there are so many noise-making “experts”. (a658) aa10 Then, as seemed inevitable, my routine was gradually postponed and postponed again, and I was often not ready for bed until one or two o’clock. But this inevitably disturbed the rest of some of my classmates (a252) aa11 But just as I was editing this article, our class actually became the emitters of noise, with another class shouting out, Stop it (a255) aa12 When I am one with my environment, I am both the receiver and the emitter of sound (a253, a258, a560, and a678) aa13 Along with the development of the times, society is progressing and our quality of life is improving, while the noise around us is also increasing (a348, a353, a649, a668, a669, and a876) aa14 There is a lot of noise around us from time to time (a239, a263, a414, a455, a541, and a600) aa15 So noise is with us all the time, it’s relevant to our lives (a284, …	A1 Noise at all times (aa1) A2 Noise is everywhere (aa2) A3 Noise cannot be avoided (aa3, aa4 and aa5) A4 People (who make noise in inappropriate situations) need to be better qualified and educated (aa6 to aa9) A5 We are both the creators and the victims of noise (aa10 to aa12) A6 Noise increases as urbanization progresses (aa13) A7 Noise is often associated with life (aa14 and aa15) A8 Noise is a mixture of multiple and single (aa16 to aa19) A9 Noise is a mixture of absolute and relative (aa20 and aa21) A10 Noise is a mixture of subjective and objective (aa22 to aa24) A11 Noise is a mixture of the simple and the rich (aa25) A12 Noise is related to sound, people and environment (aa26 to aa29) A13 Criteria for noise have uncertainty (aa30 to aa32) A14 The assessment of noise changes according to the environment (aa33 to aa35) A15 People’s perception of noise is influenced by time (aa36 to aa40) A16 People’s perception of noise is influenced by space (aa41 to aa57) A17 People’s perception of noise is influenced by visual information in space (aa58) A18 People’s perception of noise is influenced by context (aa59 to aa62) A19 Psycho-acoustic parameter characteristics (aa63 to aa65) A20 Duration of the sound (aa66) A21 Sound information content (aa67 and aa68) A22 Degree of spatial matching (aa69 to aa77) A23 Noise as defined by physical disciplines (aa78 to aa82) A24 Noise is a concept for people, and different people have different ideas of what noise is, but most people have a certain consistency in defining noise (aa83 to aa85) A25 Human perception of noise is influenced by age (aa86) …	AA1 Challenges AA2 Definition of noise AA3 Opportunities AA4 Action	AA1 Challenges (1) Sounds a: Noise at all times b: Noise is everywhere c: Noise cannot be avoided (2) Human a: People (raising their voices in inappropriate scenarios) need to promote a sense of morality and public spirit b: We are both the creators and the victims of noise (3) Environment a: Noise is often associated with life b: Noise increases as urbanization progresses AA2 Definition of noise (1) Ontology of noise concept a: A mixture of the plural and the singular b: A mixture of the absolute and the relative c: A mixture of subjective and objective d: A mixture of simplicity and richness e: Related to sound, people and environment f: Uncertainty in the criteria for noise (2) The concept of noise from environment a: Time b: Space c: Context (3) The Concept of noise from objectivity (sound) a: Psycho-acoustic parameter characteristics b: Noise as defined by physical disciplines c: Duration of the sound d: Sound information content (4) The concept of noise from subjectivity (human) a: Age b: Occupation c: Preference d: Habits e: Behavior f: State g: Environmental integration h: Psychological i: Physical j: sound expectations k: Needs l: Sensitivity m: Tolerance n: Five senses AA3 Opportunities (1) Sounds a: Noise should be seen in perspective b: Background noise has a protective buffering effect c: Steady noise helps people to fall asleep d: Noise has a reminding and warning effect e: Noise enhances fun f: Noise reduces feelings of isolation g: Noise carries memories and warmth, and carries a sense of life h: Noise has a calming effect on the emotions (2) Human a: People are adaptive to everyday noise b: People’s expectations of a quiet, natural and beautiful environment c: People’s concerns about noise are increasingly prominent (3) Technological development a: Advances in technology improved noise abatement strategies b: The active and widespread use of white noise in life AA4 Action (1) Sounds a: Classifying noise pertinently b: Controlling the loudness of sound c: Changing noise into positive sound in proper environment d: Evaluation of sound sources that can be easily identified (2) Human a: Shielding noise from the perspective of human vision b: Controlling noise from a policy rules perspective c: Limiting noise from the perspective of moral constraints d: Shielding noise from the perspective of human self-regulation and protection e: Focusing on noise from the perspective of vulnerable target groups f: Assessment of impressionable behaviors g: Characterization of susceptible states (3) Environment a: Reducing noise impact from the perspective of physical equipment and materials b: Reducing noise impact from the perspective of spatial planning and design c: Analysis of locations with high noise perception frequencies d: Evaluation of times of day when noise perception is high
Initial data collection	894 items	527 items	67 items	4 items	Generating the substantive theory

## Results

The above-mentioned grounded theory process yielded four main categories (Table 1): challenges (AA1), definitions of noise (AA2), opportunities (AA3), and action (AA4). This included internal factors, such as noise definitions (AA2), which provided a clear understanding of what noise is, and noise-related factors from the public perspective. External factors were also included, reflecting the challenges (AA1) and the opportunities (AA3) brought about by the changing times, leading to practical measures (AA4) for noise control. Details of the links between the various categories are discussed later in this section.

### The current noise challenges

The main category AA1 challenges were divided into three perspectives: sound, human, and environment.

#### Sounds

1. Noise is always present, as stated in aa1. “Noise is always present with humans. We are familiar with the word noise, which is ever present in our daily lives. Noise is often overlooked, yet it is present in every second of our daily lives…”
2. Noise is ubiquitous, as stated in recording aa2. “Noise is everywhere, but to put it in another way, having noise in empty, silent places is pointless. In our daily lives, noise is practically ubiquitous…”
3. Noise is inescapable. An interviewee in recording aa3 observed that “the world has two sides, and so does sound. I hate noise, yet I know I cannot escape it because I live in such a noisy environment. God has given us the sense of hearing, which is both a gift and a test. What should I do? I believe that noise is inevitable in today’s environment…”

#### Human

1. People who raise their voices in inappropriate scenarios should promote a sense of morality and public spirit. In recording aa7, an interviewee mentioned, “I believe that most of the noise is caused by a lack of public spirit. …they do not care about the health and feelings of others.”
2. Humans are creators and victims of noise. “I was once surprised to find that I had gone from being the one who hated noise to being a noisemaker. I had subconsciously become an emitter of noise.” As recorded in aa12.

#### Environment

1. Noise level increase as urbanization progresses. In recording aa13, an interviewee stated that “With the progress of society, cities are developing. Industrial production, transportation, people’s daily lives, and various recreational activities in cities have all expanded, and environmental noise has become increasingly serious.”
2. Noise is often associated with life. “Noisy environments are actually quite common in life. Therefore, noise is always with us, and it is relevant to our lives” (aa14 and aa15).

### Definition of noise from a popular perspective

The definition of noise AA2 is the core category and is divided into four parts: (1) the ontology of noise concept, (2) the concept of noise from the environment, (3) the concept of noise from objectivity (sound), and (4) the concept of noise from subjectivity (human). Perceiving sound as noise is a result of the complex interactions among several factors including sound, environment, and humans. See below for a detailed analysis.

#### Ontology of noise concept

1. Noise exists both in the plural and singular forms. As described in the interviews, “The definition of a noisy environment is multifaceted. Noise is a very broad concept…Sources of noise are relatively diverse. When we think of noise, we first think of broad-defined sounds, which is a slightly one-sided definition. If only the abrupt and obvious are referred to as noise, then this is only the tip of the iceberg floating on the surface of the water” (aa16–aa19).
2. Noise is a mixture of absolute and relative. As the interviewee in recordings, aa20 and aa21 suggested, “I think of noise as relative, rather than the usual sounds that are considered bad. No sound is absolute noise, and no sound is ever noise. In my opinion, noise can be divided into relative and absolute noise.”
3. Noise is subjective and objective. As stated by the interviewee in recordings aa23 and aa24, “Noise is the product of a combination of the subjective and the objective. However, noise has multiple meanings, both physical and psychological.”
4. Noise is a mixture of simplicity and richness. When asked what noise is, an interviewee was recorded (aa25) saying, “This is an interesting question, and each definition of noise has unique characteristics. The word noise is simple but extremely rich in meaning”. Noise is both obvious and evasive (Thompson, 2017).
5. Noise is associated with sound, humans, and the environment. As mentioned by the interviewee in recording aa26, “The definition of noise by different subjects is labeled differently with time and scenario. The determining factors in whether a sound becomes noises are the person and the environment. Therefore, the definition and evaluation of noise can be somewhat ambiguous…”
6. Uncertainty in noise criterion. The recording aa30 mentioned that “I think there is no rigid definition of noise. There is no definitive definition of noise in any aspect of life other than in physical terms”. It is, therefore, clear that the criteria for noise are influenced by a variety of complex factors, and that remain unclear.

#### The concept of noise from the environment

Noise is related to the environment. In this study, the recordings aa34 and aa35 stated “Whether the same sound can be considered noise is highly dependent on the environment in which it occurs. The assessment of noise changes according to the environment”. This includes time, space, and context. Perceiving sound as noise is a result of a complex interaction of factors, including the environment.

1. The perception of noise in the environment is associated with the time factor. Interviewee (recording aa37) stated, “If a faint sound that is not noticeable during the day is heard before going to sleep, it is amplified many times …” In particular, the interviewee (recording aa36) added, “During holidays, there is a lot of activity in crowded pedestrian streets, shopping malls, and restaurants, and having a lively atmosphere is acceptable.”
2. The perception of noise is influenced by the space factor in the environment. As described by the interviewee in recording aa42, “There is always Russian music playing on Central Avenue… enhancing the experience of visiting the city. By contrast, if Russian-inspired music is played in the study rooms, there is no doubt that even the most beautiful music becomes noise.” The concept of space can be defined as what an individual perceives and interacts with objectively through the connection that he or she forms with the area; each of the above showed specific responses to space based on their own and potential entrants (Akyildiz, 2020).
3. The perception of noise is related to the context factor in the environment. People’s perception is determined by the actual context (Orhan and Yilmazer, 2021). For instance, sound becomes music when placed within an appropriate context (Duffy, 2009). The interviewee in recording aa60-aa61 made a comparable point, “Some similar sounds affect me differently in different situations. We cannot conclude whether a sound is noise or not by taking it out of its specific context”. The shape of reference that determines perception is determined by the actual stimulus context (Schulte-Fortkamp et al., 2007).

#### The concept of noise from objectivity (sound)

It was found that there are five components of noise from objectivity (sound) perception: psycho-acoustic parameters of sound, noise as defined by the physical discipline, duration of the sound, and sound information content. Perceiving sound as noise is a result of a complex, dynamic process that includes sound.

1. Noise has been shown to be related to the psycho-acoustic characteristics of sound, which include roughness, as the interviewee in recording aa63 mentioned, “Noise is a noisy and confusing sound. Noise is produced when a variety of different dominant sounds (three or more) are present in a space at the same time for the person hearing the sound”. Pitch strength. The aa64 recording that: “Noise is… uncommon frequency sounds that are unattractive background sounds that are chaotic and floating. What I think of as noise is, on the one hand, a very unstable sound, with highs and lows”. For loudness, the interviewee (recording aa65) stated that, “People still accept sounds at different decibel levels to a similar degree, with sounds above 70 dB(A) generally considered to be noise”. As noise exposure increases (via longer exposure and/or higher sound levels), the risk for cochlear injury and hearing loss increases (Le Prell et al., 2020). As mentioned in the interview: “Noise can cause physical discomfort and illness in our bodies. Noise levels above 90 dB(A) can definitely damage a person’s hearing. (aa111 and aa113)”. In terms of sharpness, the interviewee in recording aa66 said, “I used to dislike the upper mathematics teacher because her voice was already sharp …which was very harsh”.
2. Noise as defined by physical disciplines. The recording aa78–aa82 stated that; “I learned that noise, as defined in physics, includes resonance at ultra-high frequencies, all irregular signals, irregular vibrations, decibels above a certain level, and irregular sound waves.”
3. The perception of noise is related to the duration of a sound. According to the interviewee in recording aa67, it is clear that “noise is usually continuous, persistent, and compels people to care about it, which has to do with the duration of the sound”. For example, “If the person next to you in the library just says something, it is quite acceptable, but if it is loud all the time, he risks drawing sideway glances from the whole library, and long and continuous noise can be more offensive and uncomfortable.”
4. Interviewees in recordings aa68 and aa69 stated that the perception of noise is related to the sound information content: “…sounds are more or less linked to the information behind them. For example, when we disagree with the speech of a person… then their voice can be considered noise to us”. “The buzzing of a mosquito, for example, is neither loud nor harsh, yet it can cause strong negative emotions because the person subjectively associates the buzzing with a mosquito bite.” Certain sounds such as those of mosquitoes and some disapproving words, can be annoying.

#### The concept of noise from subjectivity (human)

Research evidence has linked noise to people. As stated in the interviews (aa83–aa85): “noise is a concept for people. Only in human society does noise exist. However, despite this subjectivity, most people have a consistent definition of a noisy environment. Even if it is the same sound in the same environment, different people will have different answers as to whether it is noise or not”. Age, occupation, preferences, habits, behavior, status, environmental integration, psychology, sound expectations, needs, sensitivities, tolerances, and five senses have all been linked to defining noise. Therefore, perceiving sound as noise is the result of a complex, dynamic process that includes humans.

1. As described through the interviews (aa86), the perception of noise is related to a person’s age; most people felt that the older they got, the less they accepted noise and the more strongly they felt it. “I feel that as I get older, I become more resistant to noise, the interviewer says. Before I treated noise as just a bit annoying, but it was acceptable. Now I get very angry when I hear noise…My grandparents, for example, are older and prefer a quieter environment…” This is because ageing is associated with increased sensitivity to noise (Du, 2019).
2. The perception of noise is related to a person’s occupation. Different professions pay different attention to and have different feelings about noise, as described in (aa88): “Concern about noise is higher than the general population due to occupational attributes (relevant researchers)”. “Those who work in quiet indoor offices, such as programmers, are prone to the effects of harsh sounds and may be more sensitive to noise.” Noise interferes with attention and affects performance on cognitive tasks (Kjellberg et al., 1997; Moradi et al., 2019). Therefore, noise control in offices where mental work is the focus is particularly important. In addition, excessive noise exposure at work may lead to auditory health effects, such as occupational noise-induced hearing loss (Zainal Abidin et al. 2018). At the same time, exposure to occupational noise, such as in the construction industry, weakens noise sensitivity and perception of noise effects and deserves our attention (Chong et al., 2022).
3. The perception of noise is related to a person’s preference. As stated in the interview (aa91), “When I drag my mother to rock concerts, she complains about the noise. It’s loud and she doesn’t like it. For me, it’s superb and I really like it”. Soundscape evaluation is part of the study of sensory esthetics, and all esthetic issues involve preference. Esthetics also includes the ability to discern or judge. People evaluate the same environment differently and react differently as a result of their individual preferences (Yang and Kang, 2005).
4. The perception of noise is related to a person’s habits. For example, in the interview (aa105) it was stated that “Some people are taught not to make noise when they eat and grow up with an aversion to the sound of their mouths, which is obviously noise to them, but for other groups who believe that the sound of eating means I am eating well, the sound of their mouths during eating is physically and emotionally pleasing”.
5. The perception of noise is related to a person’s behavior. As stated in the interview (aa135), “The sensitivity to noise varies with what we are doing, and the definition of noise should be different for different behaviors”. For example, interview (aa170) mentions that “when we are studying or resting, all sounds that can be clearly heard, whether they are pleasant or not, are considered noise… while when we are exercising, we often find the natural ambient sound not loud enough, or even like to play some punk rock music with strong drums and dense rhythms at a high volume”.
6. The perception of noise is related to a person’s mental state. As described in the interview (aa173), “It has been observed that people tend to ignore distracting noises when they are concentrating on a certain state… When a person is initially in an office state, he can easily feel annoyed and dissatisfied because of the distracting noises around him, and as he gradually immerses himself in his work, he can no longer easily feel the presence of these noises.”
7. The perception of noise is related to a person’s environmental integration. The environment is highly integrated and less influenced by noise. When one is involved in the current environment, he/she ignores the surrounding noise, whereas one is not involved in the current environment, the opposite feeling arises. For example, as described in the interview (aa199 and aa214): “In a public space, a group of people talking to each other and a person doing their own thing, to the person talking their conversation is nothing, to the other person doing their own thing their conversation is noise. When I am interacting with my friends, I don’t find the noise of people talking and laughing with me at all”. We are both the emitter and the victim of noise. Being the source of noise ourselves, we often ignore the noise we produce. However, we are disturbed by the noise produced by others.
8. The perception of noise has been linked to a person’s psychological state. Noise and psychology interact, and when psychological conditions are poor, one becomes more critical of sound and more sensitive to noise. For example, “when I am irritable and unhappy, I am more critical of my surroundings and even sounds that I would not normally pay much attention to become noise… In everyday life…whether it is judged as noise or not often depends on the subjective state of mind” (aa130 and aa132). Noise and psychology interact and may form a vicious circle. As described in the interview (aa134), “these sounds that become more emotionally distressing the more we care, lead to a more depressed mood and the more noise we hear, which then creates an emotionally vicious circle”. Emotion, a key psychological antecedent of cognition, can frequently influence or even overpower our right conclusions (Yang et al., 2018). Humans use emotion processing as the primary channel for shaping ideas and decisions (DANIEL and Review, 2003).
9. The perception of noise is related to a person’s physical condition. For example. “Being unwell and the sound of your roommates talking can make you feel uncomfortable” (aa108). When physiological conditions are poor, the perception of noise and discomfort increases. This may be because people are more vulnerable when they are physically uncomfortable which makes them more susceptible to noise disturbance compared to healthy individuals. Thus, there is a need to focus more on the physical discomfort and to actively avoid noise interference.
10. The perception of noise is related to a person’s sound expectations. People expect more sound at certain times and occasions and when they are engaged in certain behaviors. As mentioned in the interviews (aa194–aa197), “when studying in the library and needing a quiet environment, the expectation at this time is that the sound is quiet, but the music that I normally enjoy listening to becomes noise because it does not match the environment I need at the moment”. Sound expectation is part of a framework of cognition and emotion when perceiving soundscape contexts. Expectation is related to an individual’s experiences and encompasses factors such as personal beliefs, perspective, ideals, values, emotions, and mental models. Expectations strongly influence perception.
11. The perception of noise is related to a person’s needs, as described in the interview (aa146 and aa147), “When a sound does not satisfy my needs, I feel that the sound is noise. Like myself, I like to listen to songs when I am going to study. I do not really understand why I concentrate more when I am listening to a song when it is obviously louder than my classmates’ voices. Maybe it’s psychological, or maybe the songs are what I want subjectively”.
12. The perception of noise is related to a person’s sensitivity, as described in the interview (aa182 and aa189), “I know people who are quite sensitive to sound and can’t sleep at the slightest noise, and others who can sleep even when thunder rolls through the sky. People’s sensitivity to noise also varies from person to person…”The perceived annoyance of noise has a great difference among individuals with different noise sensitivities (Di et al., 2022). Noise-sensitive individuals are more susceptible to noise-induced annoyances (Miedema and Vos, 1999).
13. The perception of noise is related to a person’s tolerance. People’s noise tolerance levels vary, as stated in the interview (aa190): “I think everyone has a different level of tolerance and acceptance of noise, which I personally believe is inextricably linked to one’s occupation, physiological performance, living environment, habits and personality”. As stated in the interview (aa190), “noise should have different standards and tolerances for different people. I have a higher tolerance for the noise created by people I know and am close to, and a lower tolerance for people I don’t know or don’t like”.
14. The perception of noise is associated with a person’s five senses. As described in the interview (aa152), “to be judged in conjunction with sight, taste, touch and smell, specific to the problem”. The assertion “we unknowingly can use all senses information to assess sounds” emphasizes the influence of other sense sensations on sound perception. In addition, ‘Audio–visual interaction can significantly influence the outcome of the definition of quiet areas (Li and Lau, 2020)”. Greater attention to the visual landscape can lead to reduced hearing perception and vice versa (Southworth, 1969). Similarly, the presence of fragrance enhances people’s evaluation of traffic noise and improves auditory and olfactory satisfaction, demonstrating the interaction between hearing and smell (Ba and Kang, 2019a). Besides, there is a masking effect between the auditory and olfactory senses (Ba and Kang, 2019b). Different types of background sounds can influence taste and flavor perception. In addition, background noise affects sensitivity to taste perception (Rahne et al., 2018). For instance, higher-pitched background music can elicit stronger associations with the sweetness dimension, thereby increasing the perception of sweetness (Stafford et al., 2012). Pleasant sound and audio-visual stimuli can enhance the taste of food (Kantono et al., 2016a, 2016b). On the other hand, temperature sensation is traditionally regarded as a highly discriminative tactile capacity allied to the somatosensory system (Craig, 2009). In real life, thermal and acoustic environments have an important influence on the subjective and physiological responses of people, which together affect their comfort (Guan et al., 2020b). Noise does not affect thermal sensation, but affects thermal comfort, while temperature affects acoustic sensation (Nagano and Horikoshi, 2005; Yang and Moon, 2018). Noise should be controlled in a high-temperature environment to ensure comfort (Guan et al., 2020a).

### The noise opportunity along with time change

The main category AA3 Opportunities was divided into three viewpoints, i.e., sound, human, and technological development.

#### Sound

(1) Noise should be viewed in context: “Noise is not always harmful. I believe it should be viewed in a dialectical way. For instance, white noise, which is beneficial, should not be rejected, but tolerated” (aa216 and aa217). In general, noise is harmful since it renders organisms less flexible and potentially causes gene mutation. Nonetheless, accumulating evidence suggests that noise helps in stress response, metabolic activity, development, cell cycle, circadian rhythms, and aging, as well as regulates cellular functions (Zhou et al., 2022).

(2) Background noise is a protective buffer: According to the interviews (aa299), “…just as we are unaccustomed to a 0 dB environment, it is frightening to be in a condition of absolute silence. If in a relatively quiet environment, a loud noise is suddenly generated… it is easy to be frightened when you are unprepared. This is when the noisy environment that we normally take for granted becomes a kind of protection for us again”. All the disturbing sounds in the environment can be muffled by monotonous sounds, including white noise, which is a combination of constant sound frequency variations within the environment (Umbas et al., 2021).

(3) Noise induces sleep: As described in the interview (aa23), “…when the snow-plows were roaring in the winter evenings, I was undisturbed by the sounds because they were stable. On the contrary, it even helps me sleep very much.” This is because humans easily get used to steady noise or repetitive monotonous noise. Steady noise may induce sleep, whereas intermittent noise does not (Suzuki et al., 1991).

(4) Noise acts as a reminder and a warning: As described in interviews (aa233 and aa234), “…The air-raid siren on China’s September 18 anniversary was deliberately special, attention-grabbing, and slightly uncomfortable sound… aimed striking the heart of the people, awakening inner emotions, warning and spurring the present generation not to forget the war.” This shows that although noise evokes discomfort, it generates emotions within people.

(5) Noise elicits fun: “Within reasonably normal limits, noise is interesting” (aa237). For example, the noise was used as an artistic and esthetic resource, a source of music, and to create new sound sensations in the 20th and 21st centuries (Thompson, 2017).

(6) Noise reduces feelings of isolation: “…late at night…the sound of cars whizzing by on the road is the only reason I don’t feel alone…” (aa235 and aa236). Silent individuals feel uncomfortable. Thus, the concept of quiet zones, currently emphasized, warrants further exploration and refinement. This is because a good acoustic environment is not defined by the quiet standard alone.

(7) Noise transports memories, warmth, and a sense of life: A soundscape positively or negatively affects the way of life. Soundscapes attempt to recreate historical scenes (Akyildiz, 2020). As part of a soundscape, noise carries memories and warmth, with a sense of passionate living. As stated in interviews (aa222 and aa223), “Perhaps at home, my mother’s nagging is something I wish would go away, but after leaving home… I wish I could hear her once more telling me to get up in the morning and bring my homework to school…” Noise brings a sense of life noticeable at certain times. For instance, during the new crown epidemic and festive season, too much silence brought a stronger sense of loneliness, whereas noise reflects a sense of life and joy. Interview (aa220 and aa221) states: “Every New Year’s Eve, the noise market and roar of firecrackers bring joy and warmth. The school was closed due to COVID-19… and I was often unable to leave the school for long periods, and whenever I was unblocked to go to the food market outside the school…I didn’t feel the noise, I just felt a sense of hope and enthusiasm for life”.

(8) Noise has an emotionally calming effect: Interviews (aa230 and aa231) revealed that white and pink noise from nature calms. The interviews were as follows: “Some white noise, which is the sound of nature, like the wind, rain, birdsong, and so on, is an ambient sound for us, and such sounds make us feel settled.”

#### Human

(1) People adjust to daily noise: As mentioned in the interview (aa238): “Noise pollution is a daily occurrence and most people do not see it as a problem; most of the noise we hear every day is related to everyday activities, so people are used to it.” Although most people are habitually exposed to noise, the habituation extent varies among individuals (Basner et al., 2014).

(2) People expect a quiet, natural, and beautiful environment: As stated in the interview: “The demands on the quality of the surrounding environment will also be more demanding… Nowadays, people are increasingly looking for “quiet”. There has always been a picture of birds and flowers, paths in the fields, two or three friends and a glass of wine…” (aa239–aa244). Similarly, people demand soundscapes having simple sound developments with messages of peace and joy (Liu and Kang, 2016).

(3) The concerns about noise are increasingly prominent: As the recording states: “The noise problem has become more and more serious in recent years and more and more people are paying attention to it. Noise pollution can be harmful and needs to be taken seriously” (aa247–aa249). There is a growing concern for environmental quality, specifically noise and air issues (Chiarini et al., 2020). Air issues have received a lot of attention in recent years; however, noise issues have been neglected (TheLancet, 2014).

#### Technological development

(1) From an environmental standpoint, technological developments have reduced noise pollution. Interview (aa250) states that “… I think noise can be dissipated. But the prerequisites for this would be very many. For example, it would require modern technology to develop to a point where invisible earplugs could be invented with 100% noise dissipation…” (2) Furthermore, active and widespread use of white noise provides new ideas for the rational use of noise. The interviews (aa251–aa253) state that “…white noise is noise whose power spectral density is constant throughout the frequency domain. For example, some learning apps have a ‘white noise background to help people focus better.” Several studies have investigated devices producing white noise to offset and minimize the effects of environmental noise in clinical and laboratory settings. Since white noise is actively used in everyday life, several studies have focused on the impact of white noise on humans. Consequently, the understanding of white noise in the physical sense and white noise by the general public is not similar. Therefore, there is a need to investigate a definition of white noise based on the general public.

### Noise problem-solving measures

The main category AA4 Action was divided into three viewpoints, i.e., sound, human, and environment.

#### Sounds

(1) Noise classification should be specific: As stated in the interview (aa255), “I believe that the classification of noise should be more focused. The criteria for judging noise today are uncertain…” Despite being unpopular, noise should be classified and controlled from a qualitative perspective in the future (Schafer, 1996).

(2) The loudness of the sound can be controlled: As stated in the interview (aa254): “…after a certain dB(A), noise has an adverse effect on what we are doing…”. From a quantitative standpoint, controlling sound decibels is easy (Schafer, 1996). Based on the noise source, the WHO recommends different control recommendations for environmental noise thresholds. For instance, noise levels generated by road traffic should reduce to below 53 dB L_den_; by railway traffic below 54 dB L_de;_ by aircraft to below 45 dB L_den_. Noise levels from wind turbines should be reduced to below 45 dB L_den_, and the annual average noise from all recreational noise sources combined should be reduced to 70 dB L_Aeq,24h_. Otherwise, it may have negative effects on health (WHO, 2018).

(3) Noise can be converted into positive sound in the right environment: As stated in the interview (aa256), “The most beautiful sounds can be achieved if they occur in the right environment”. The design of the ambient sound improves the attractiveness of a venue. For instance, laughter in a library is an inappropriate negative sound, but laughter in an amusement park or at a party is a positive sound driving the atmosphere.

(4) Sound sources easily identified as noise: The interview content (A56) of the main response noise sources was extracted and screened (Selection criteria are frequency ≥ 5 times) (Table 2). Traffic noise is most likely identified as a sound source and perceived when studying (7 times). Traffic noise occurs in the street, where people usually perceive traffic noise including vehicle sirens (16 times) and vehicle moving sounds (8 times). Besides, construction sound is usually felt at home (9 times), primarily at building sites (17 times) and homes (12 times). Traffic noise (41 times) and construction sound (36 times) are mentioned more than 30 times and require our attention. Details of the frequency, time, and location of the perceived sound sources are tabulated below. Based on the standpoint of sound sources, knowing the sound sources that can be considered noise allows more targeted prevention and control measures.

**Table 2:** Sound sources easily identified as noise.

Sound source	Number	Behavior and state	Place	Details
Traffic noise	41	When studying (7)	Street (6)	Vehicle sirens (16) Vehicle moving sound (8)
Construction sound	36	Staying at home (9)	Building site (17) Home (12)	
Machinery	21	Staying at home (11)	Home (11)	
Conversation	20	When sleeping (8)		Talking in a low voice (5)
Music	16			
Film and television broadcasting	9			TV sound (5)
Animal sounds	9			Dog barking (7)
Shouting	8			
Fingernails scratching on the blackboard	5	During a lesson (5)	Classroom (5)	
Sounds of playing computer games	5			
Sounds of children playing	5		Home (5)	

#### Human

**Fig. 5:**
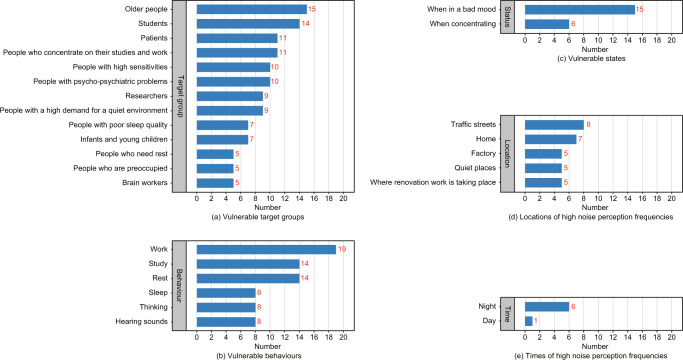
Quantification of word frequency statistics. **a** Vulnerable target groups, **b** Vulnerable behaviors, **c** Vulnerable states, **d** Locations of high noise perception frequencies, and **e** Times of high noise perception frequencies.

(1) Shielding noise from the perspective of human vision: As stated in the interview (aa60), “…I find it easier to concentrate when I am visually invisible to the source of the noise… This has inspired me to design spaces in the future in such a way as to reduce the impact of noise on people by increasing visual concealment or adding partitions”. Acoustic and visual (and other) components interact in human perception (Brown et al., 2011). Masking the sound source reduces loudness perception and noise annoyance (Masullo et al., 2021). Also, the use of color leads to different perceptions of sound loudness (Menzel et al., 2008).

(2) Controlling noise control from the perspective of policy: From the perspective of noise control, policies should be developed to control the loudness of sound, the time and place where the noise is emitted, and noise-generating behavior. As mentioned in the interviews (aa390, aa391, aa394, aa395), “The sound of car horns is prohibited. As well as limiting the working hours of construction sites. I think it is necessary to set strict noise control levels around specific buildings…to introduce regulations for this….set strict decibel levels…”

(3) Limiting noise based on moral constraints: As shown in the recordings (aa401), “I think we should be more tolerant and also abide by the rules of public places”. Morality and its role in societal life have been critical to human thought history thought since its beginnings. It is ranked first in Aristotle’s hierarchy of virtues as a good to which every human being must aspire (Mb et al., 2021).

(4) On human self-regulation and protection, it is possible to shield oneself from the noise by negotiating with its source: For example, as stated in the interview (aa376): “negotiate with your neighbor to turn down the volume of the TV.” Protect your ears from noise through simple means, “Silicone earplugs are available and these are simple and practical means to provide warning and protection when noise is coming” (aa381). This is because noise discomfort triggers the body’s defense mechanisms, hence prompting people to adopt appropriate protective behaviors to resist additional harm from noise (Park et al., 2018; Tinoco et al., 2019).

(5) Focus on vulnerable target groups and extract and screen interviews (A62) primarily responsive to them (Selection criteria are frequency ≥5 times): This is also aimed at determining the frequency of vulnerable behaviors in descending order: Older people (15 times); Students (14 times); Patients (11 times); People concentrating on their studies and work (11 times) High sensitive People (10 times); People with psycho-social problems (10 times); Researchers (9 times); People with a high demand for a quiet environment (9 times); People with Poor sleep quality (7 times); Infants and young children (7 times); People needing rest (5 times); Preoccupied people (5 times); Brain workers (5 times) (Fig. 5a). Thus, there is a need to focus on noise-sensitive people.

(6) Vulnerable behavior: Focusing on these vulnerable behaviors, the interview content (A63), which primarily responds to the behavior, was extracted and screened (Selection criteria are frequency ≥5 times) to arrive at the most vulnerable behaviors in descending order of frequency: working (19 times), studying (14 times), resting (14 times), sleeping (8 times), thinking (8 times) and hearing sounds (8 times) (Fig. 5b). Locations, where these behaviors occur, should be an area of focus when characterizing noises.

(7) Vulnerable states: The interview content (A64) primarily responding to the person’s state was extracted and screened (Selection criteria are frequency ≥5 times) to establish the susceptible states, i.e., in a bad mood (15 times) and during concentration (6 times) (Fig. 5c). Therefore, it is important to protect oneself from the intrusion of noise when in these moods or highly concentrated states.

#### Environment

(1) Reducing noise impact based on spatial planning and design: As demonstrated in the recording (aa483), “…For example, the location of hospital buildings, the layout of ward buildings, outpatient buildings and other departmental rooms, the design of doors and windows, the choice of interior and exterior building materials, and the planting of surrounding vegetation”.

(2) Reducing noise impact according to the physical equipment and materials: As demonstrated in the recordings (aa490, aa491, aa493, aa494), “We need measures that prevent harmful noise, including retrofitting roads with soundproof panels. Measures to control these noises often include the installation of silencers on cars…” Car silencers reduce noise right at the source. Noise barriers, acoustic glass, and other sound-absorbing materials are passive methods using physical structures and affect sound waves before they enter the interior of the building. These are classical, simple, and easy-to-use methods (Lam et al., 2021).

(3) Locations with high noise perception frequencies: The interview content (A67) was extracted from locations primarily responding to noise perceptions and filtered (Selection criteria are frequency ≥5 times) to show exact locations with the highest frequency of noise perceptions in order: traffic streets (8 times), homes (7 times), factories (5 times), quiet places (5 times) and locations where renovation work is taking place (5 times) (Fig. 5d). This correlates somewhat with the susceptible behaviors and states derived above, because of fixed locations that limit human behavior.

(4) Time of day with high noise perception: The time points were extracted from the interview content (A68) mainly responding to the time of noise perception and filtered (Selection criteria are frequency ≥5 times) to establish the time of night (6 times) with the highest frequency of noise perception (Fig. 5e). This may be because the night is considered resting time, and people are sensitive to noise and have higher requirements for quiet environments.

## Discussion

To understand our findings, three key points must be emphasized; the details are as follows:

1. What is the relationship between different categories?
2. What is the impact of this study on human perception of noise?
3. What is the source of inspiration for us in noise study from human perception?

### The relationship between the four categories

**Fig. 6:**
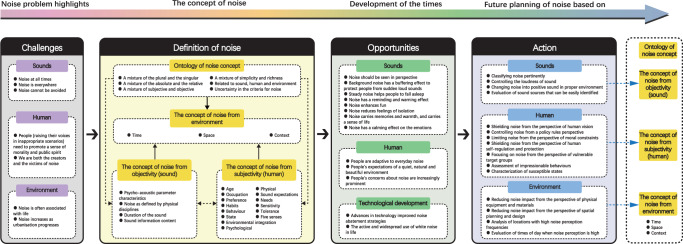
The structural links between the categories.

The first topic is the relationship between different categories, as shown in Fig. 6, ranging from challenges to measures:

1. *Challenges*: The three components i.e., sound, human, and environment (social/physical) demonstrate the importance of the noise problem and its challenges as an urgent issue that must be addressed. Notably, noise is present at all times, it is everywhere, and it cannot be avoided.
2. *Noise definition*: The definition of noise includes four components, i.e., noise concept, noise from environment perception, noise from objectivity (sound) perception, and noise from subjectivity (human) perception. The overarching concept of noise acts on the three components i.e., human, sound, and environment. Sound and humans are in an interactive relationship; both exist in and interact with their surroundings.
3. *Opportunities*: The advantages of noise based on current developments, as well as environmental changes caused by changes in human thinking and technology. Considering the positive effects of noise, noise should be viewed from the following perspective: noise carries memories and warmth, and a sense of life; background noise has a buffering effect to protect people from sudden loud sounds; noise has a calming effect on the emotions; steady noise helps people fall asleep; noise has a reminding and warning effect, noise reduces feelings of isolation; noise enhances the fun. The current social environment opportunities and technological developments enable noise abatement, as well as the active and widespread use of white noise in daily life.
4. *Action*: Plans and measures for noise are proposed based on sound, human perception, and the environment, including controlling the loudness of the sounise transformation into positive sound in the right environment; shielding noise based on human self-regulation and protection; focusing on noise from based on vulnerable target groups, noise control from the perspective of policy rules; shielding noise according to human vision; limiting noise according to moral constraints; reducing noise impact based on spatial planning and design; reducing noise impact from according to the physical equipment and materials.

### The effect of noise on human perception

**Fig. 7:**
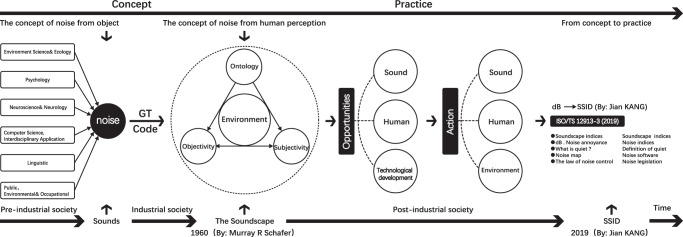
From “noise control” to “soundscape” to SSID.

As shown in Fig. 7, this focused on the progression from noise control to soundscape definition, then to the application with SSID. Figure 7 shows the concept axis (from concept to practice). The article examines noise literature, from the following disciplines: Environmental Science and Ecology, Psychology, Neuroscience and Neurology, Computer Science, Interdisciplinary Application, Linguistic and Public, and Environmental and Occupational Health. A grounded theory approach expands the definition of noise in six disciplines at the level of human consciousness. Ontology, object, subject, and environment are the four dimensions of noise. In this view, opportunities and measures of noise are explained in three dimensions, i.e., sound, human, and environment (physical/social). Eventually, there is integration and use with SSID.

Figure 7 (bottom part) shows the timeline (from 1960 when soundscapes were proposed, to 2019 when SSID was proposed). First, we looked into noise and discovered similarities with soundscape definition, both of which refer to the environment and people. The work of a musician and composer revolves around the relationship between the ear, humans, sound environments, and society. Schafer (in the late 1960s) is the pioneer of the soundscape. (Schafer, 1996). Subsequently, the International Organization for Standardization (ISO) defines this acoustic advancement with the ISO/FDIS 12913-1 2014. A soundscape is “the acoustic environment as perceived, experienced, and/or understood by a person or people in context.” Therefore, soundscape research is a fundamental shift in the field of sound evaluation (Kang and Schulte-Fortkamp, 2015). Human perception of the acoustic environment should be in the soundscape. In ISO/TS 12913-3: Version 1 (2019-12) noise is mentioned in the following areas. “Based on the tradition of environmental noise studies, the term ‘annoying’ is used instead of ‘unpleasant’. According to environmental noise assessment, noise mappings are used to evaluate the noise effects on humans. The auditory system detects small differences based on short-term memory, hence individuals rely more on characteristic noise features and patterns” (Standardization IOf, 2014). The above passage on noise from ISO/TS 12913-3: First edition (2019-12) only addresses, noise maps, and sound levels, but ignores the modeling, description, and definition of human perception of noise. Jian KANG et al. (2019) proposed the Soundscape Index which optimizes the acoustic environment, hence promoting research on the soundscape. The “Soundscape Indices” (SSID) i.e. SSID may accurately describe levels of human comfort by considering psychological, (psycho) acoustical, neural and physiological, and contextual factors. As a result, SSID integrates alongside (and eventually replaces) sound level metrics in existing (international) regulations, shifting the focus away from sound insulation toward a more comprehensive view (Kang et al., 2019). The soundscape approach and SSID help in understanding the process of perceiving the acoustic environment in the human environment and improving sound quality in the human environment.

In conclusion, our findings expound on the definition of soundscape by refining the concept of noise at the level of human consciousness and providing a qualitative research direction to improve the ISO standard.

### Inspiration for investigating noise from a human perception perspective

The importance of studying noise from a human perception is that noise not only have a negative effect, but also a positive effect in some cases. Therefore, it is important not to completely remove it but exploit its positive effects in appropriate situations. This view coincides with the soundscape approach, which addresses issues related to the sound environment from a sound perspective, in which sound is not treated as waste but as a resource (Kang, 2019). This study widens the definition of noise at the human cognitive level, thus improving the definition of noise.

Noise not only affects several of people’s lives in terms of psychological, physiological, and behavioral aspects in addition to studies, recreation, and rest. People’s subjective perception of sound is the soundscape, and perception contains both positive and negative components. Generally, the negative part of soundscape is considered noise, which is dependent on the listener’s experience of the sound and the environment. Schafer believed that the soundscape can demonstrate a society’s illness or well-being. A soundscape that is ordered and peaceful reflects a society that is ordered and harmonious, whereas a soundscape that is disordered and discordant causes social disorder and disharmony (Schafer, 1996; Thompson, 2017). This study shows that it is important to recognize the positive effects of noise, or how they can be applied in scientific studies. Thompson suggested that noise can and does sometimes have a beneficial effect and the negative effects of noise are often exaggerated (Thompson, 2017). Our understanding of noise in terms of human perception and utilization of a broader soundscape approach can help move from (traditional) noise control to designing acoustic environments that will improve environmental quality.

## Conclusion

The study studied the concept of noise from the human perception. Historical noise complaints were reviewed by analyzing studies on noise (VOSviewer) and relevant bills. Systematic qualitative analysis of user-centered noise and grounded theory interview methods were adopted. In addition, the basic understanding of noise from the perspective of the public was explored to uncover all possible understanding of noise from the user’s point of view. Our results present a comprehensive understanding of the noise environment, and highlights the importance of examining noise from the perspective of the public, which will ensure effective control of noise.

1. *Four categories are identified in this study*: Challenges, the definition of noise, opportunities and action. By analyzing the external factors of noise: challenges and opportunities, as well as internal factors: definitions of noise, action, an implementable strategy model for noise control was established from a public perspective.
2. *Challenges*: As urbanization progresses, noise levels increase, and we are all creators and victims of noise. Noise is everywhere, every time and thus unavoidable. Therefore, it is important to understand how people perceive the noise to develop effective noise control strategies.
3. *Definition of noise*: Perceiving sound as noise is a result of a complex and dynamic process which include sound, the environment, and humans. The environmental aspects include time, space, and context. The sound aspect includes duration of the sound, content of the sound message and other factors. On the other hand, the human aspect of noise includes factors such as age, occupation, tolerance, and five senses.
4. *Opportunities*: Noise not only have negative effects, but also positive effects in specific situations, which are related to how people perceive sound in their environment. The positive effects of noise include carrying memories and warmth, providing a sense of life, providing a buffering effect that protects people from harmful effects of sudden loud sounds, calming emotions, reminding, and warning people, as well as reducing feelings of isolation. On the human side, although people are adapting to noise, they are more concerned about noise issues and aspire to a better life and a more beautiful environment. Recent technologies have shown good performance in reducing noise and white noise is now an active and widespread part of life. In recent years, focus has shifted from noise control to noise application.
5. *Action*: Noise needs to be classified and its loudness needs to be controlled, which will turn it into a positive sound in the right environment. From the human side, noise can be shielded from human vision by focusing on susceptible behaviors such as working, studying, resting, sleeping, thinking, and listening to sound. Besides, this can be achieved by focusing on susceptible states, such as when in a bad mood, when concentrating, as well as on the vulnerable target groups such as older people, students, patients, researchers, people with poor sleep quality, infants, and young children. From the environmental aspect, the impact of noise can be reduced from the perspective of physical equipment and materials, and from the perspective of spatial planning and design. It is also important to focus on locations with high noise perception frequencies, such as traffic streets, homes, factories, quiet places, and places where renovation work is carried out. In addition, there should be focus on times of the day when noise perception is high, especially at night.

This study addresses three components, i.e., human, sound, and environment. Sound and human form an interactive relationship in the environment. The soundscape approach together with its indices (SSID) is appropriate in achieving a better understanding of this process. Moreover, our study refines the concept of noise at the level of human consciousness. The current study investigated noise control, soundscape definition, integration, and SSID application. Findings from this study are expected to guide future improvement on the definition, control, and utilization of noise, moving from (traditional) noise control to modern design of acoustic environments, thereby increasing quality of the sound environment.

Conventional perceptions may have led many people to believe that noise is negative. We have come up with the theory that “ Perceiving sound as noise is the result of a complex dynamic process that includes sound, the environment, and humans”. Given that noise has both positive and negative aspects, what should positive noise be called? What are personal preferences for terms and how are they defined? These are questions that deserve to be explored in future.

## Data Availability

Datasets generated during the current study (interview transcripts) are not publicly available due to a confidentiality agreement signed with interviewees. Nevertheless, they can be requested from the corresponding author on reasonable request.
